# Hemispheric Asymmetry in TMS-Induced Effects on Spatial Attention: A Meta-Analysis

**DOI:** 10.1007/s11065-023-09614-2

**Published:** 2023-09-22

**Authors:** Ting Wang, Tom de Graaf, Lisabel Tanner, Teresa Schuhmann, Felix Duecker, Alexander T. Sack

**Affiliations:** 1 Department of Cognitive Neuroscience, Faculty of Psychology and Neuroscience, Maastricht University, 6200 MD Maastricht, the Netherlands; 2 Maastricht Brain Imaging Centre, Maastricht, the Netherlands; 3 Department of Psychiatry and Neuropsychology, School for Mental Health and Neuroscience, Maastricht University Medical Centre+, Brain+Nerve Centre, Maastricht, the Netherlands; 4 Centre for Integrative Neuroscience, Faculty of Psychology and Neuroscience, Faculty of Health, Medicine and Life Sciences, Maastricht University, Maastricht, the Netherlands

**Keywords:** Hemispheric Asymmetry, Transcranial magnetic stimulation, Spatial attention, Landmark, Line bisection, Meta-analysis

## Abstract

Hemispheric asymmetry is a fundamental principle in the functional architecture of the brain. It plays an important role in attention research where right hemisphere dominance is core to many attention theories. Lesion studies seem to confirm such hemispheric dominance with patients being more likely to develop left hemineglect after right hemispheric stroke than vice versa. However, the underlying concept of hemispheric dominance is still not entirely clear. Brain stimulation studies using transcranial magnetic stimulation (TMS) might be able to illuminate this concept. To examine the putative hemispheric asymmetry in spatial attention, we conducted a meta-analysis of studies applying inhibitory TMS protocols to the left or right posterior parietal cortices (PPC), assessing effects on attention biases with the landmark and line bisection task. A total of 18 studies including 222 participants from 1994 to February 2022 were identified. The analysis revealed a significant shift of the perceived midpoint towards the ipsilateral hemifield after right PPC suppression (Cohen’s *d* = 0.52), but no significant effect after left PPC suppression (Cohen’s *d* = 0.26), suggesting a hemispheric asymmetry even though the subgroup difference does not reach significance (*p* = .06). A complementary Bayesian meta-analysis revealed a high probability of at least a medium effect size after right PPC disruption versus a low probability after left PPC disruption. This is the first quantitative meta-analysis supporting right hemisphere-specific TMS-induced spatial attention deficits, mimicking hemineglect in healthy participants. We discuss the result in the light of prominent attention theories, ultimately concluding how difficult it remains to differentiate between these theories based on attentional bias scores alone.

## Introduction

Hemispheric asymmetry is a key concept in the functional architecture of the brain and plays a core role in many spatial attention theories. This is also, and maybe even primarily, based on the hemispatial neglect phenomenon, a syndrome where patients struggle to allocate attention to, or even simply detect, stimuli in the left hemifield. It is more common and severe after right hemisphere damage (Beis et al., 2004; Corbetta et al., 2005; Suchan et al., 2012). This consistent evidence has inspired different ideas about the contribution of the right versus the left hemisphere to attentional processing (e.g.Mennemeier et al., 1997; Mesulam, 1981). An early intuitive idea based on this functional asymmetry seen in neglect patients is that the right hemisphere of the human brain is dominant for relevant attention processes. This right hemispheric dominance in attention (e.g., Shulman et al., 2010) would thus represent a general principle of the brain similar to the left hemispheric dominance in language processing regions (Geschwind, 1972).

There are two widely supported theories of attention, the Heilman’s hemispatial theory and the Kinsbourne’s interhemispheric competition theory (Duecker & Sack, 2015). Both theories conclude that the right hemisphere causes more pronounced functional attention effects; however, they both explain this asymmetry very differently by accounting for the contribution of each hemisphere to attentional control in fundamentally different ways. The Heilman’s hemispatial theory postulates that the right hemisphere is not necessarily “stronger,” but rather has an expanded function. Namely, the right hemisphere shifts attention to both visual hemifields, while the left hemisphere is only able to shift attention to the right visual hemifield (Heilman & Van Den, 1980). In this model, left parietal lesions can be compensated for by the right hemisphere but not vice versa. Kinsbourne’s interhemispheric competition theory, on the other hand, postulates that the left hemisphere is dominant, but that, importantly according to the interhemispheric competition model, both hemispheres induce attentional bias towards their respective contralateral visual hemifield, exerting reciprocal inhibition over one another to maintain system balance (Kinsbourne, 1977). In this theory, the left hemisphere–induced bias towards the right hemifield is somewhat stronger and once disinhibited after right hemispheric lesion (loss of interhemispheric balance), this stronger functional spatial attention bias towards the right side of space causes left hemineglect.

Additionally to neuropsychological evidence of hemispheric asymmetries in the functional relevance of left versus right parietal cortex, a more rigorous and controlled experimental investigation of these asymmetries in healthy volunteers using neuroscientific research tools is paramount. Although extensive neuroimaging work on human visual attention has investigated the involvement of the right and left hemisphere during the execution of various attention tasks (e.g., Corbetta & Shulman, 2002; Driver et al., 2004; Serences & Yantis, 2006), standard neuroimaging studies are not sufficient on their own to address hemispheric differences (Ruff et al., 2009). Transcranial magnetic stimulation (TMS), as a functional intervention, allows the temporary modulation of local neural activity in healthy individuals (Pascual-Leone, 2000), revealing a subsequent inability to perform a particular behavior, TMS can thus be regarded as a unique research tool for the investigation of causal structure–function relationships (Sack, 2006). TMS modulates behavior depending on the used protocols (Silvanto & Muggleton, 2008), but in general terms, TMS is often conceptualized as inducing a change of excitability (offline protocols) or a disruption of ongoing processing (online protocols) (Veniero et al., 2016). Several studies used TMS to induce “virtual lesions” in parietal nodes of the visuospatial attention network in healthy volunteers to induce transitory biases simulating symptoms of spatial neglect (Babiloni et al., 2007; Chambers et al., 2004; Esterman et al., 2007; Fuggetta et al., 2006; Harris et al., 2008; Koch et al., 2005; Rounis et al., 2007; Rushworth et al., 2001; Sack, 2010). Importantly, inducing neglect-like attentional deficits in healthy volunteers using TMS in a well-controlled laboratory settings holds the promise of gaining more specific insights into hemispheric asymmetries in attention (Salatino et al., 2014; Szczepanski & Kastner, 2013). While the exact nature of the effects induced by TMS may not directly mirror the deficits observed in neglect patients, studying the effects of TMS on spatial attention provides valuable insights into the underlying neural mechanisms and potential functional contributions of different brain regions. By manipulating neural activity in specific brain areas, TMS allows us to investigate the causal relationship between brain regions and cognitive processes. By exploring the relative effects of stimulating the left versus right hemisphere, we contribute to the understanding of the hemispheric dominance in spatial attention and provide insights into the predictions of attention theories.

The line bisection (LB) and landmark (LM) tasks have played prominent roles in assessing attentional bias in neglect patients as well as TMS-induced attentional bias in healthy volunteers (Fierro et al., 2006; Giardina et al., 2012; Mahayana et al., 2014). Both tasks require individuals to judge the midpoint of a line. However, in the LB task, participants mark the judged midpoint themselves, whereas in the LM task, the line is pre-bisected and participants have to judge whether this bisection is correct (Cicek et al., 2009; Learmonth & Papadatou-Pastou, 2022; Strappini et al., 2023). Patients with right posterior parietal lesions tend to judge the middle-point of the line to be slightly right of true center, indicating a rightward shift of attention (Chatterjee et al., 1999; Verdon et al., 2010). A leftward shift is substantially less likely to emerge following left parietal damage (Karnath & Rorden, 2012). Several studies have successfully employed the LB and LM task also in healthy volunteers using TMS to inhibit their left and/or right posterior parietal cortex (PPC; left PPC: LPPC; right PPC: RPPC) and assessing the behavioral consequences on attention task performance in a controlled experimental setting (Bagattini et al., 2015; Brighina et al., 2002; Cazzoli & Chechlacz, 2017; Ellison et al., 2004; Giglia et al., 2015; Salatino et al., 2019; Salatino et al., 2014; Szczepanski & Kastner, 2013). However, a systematic investigation of the existing TMS literature is still missing. TMS effects are often small, studies notoriously underpowered, and findings often not replicable across laboratories (Gilmore et al., 2017). The question, thus, whether TMS is indeed capable of reliably producing significant spatial attention effects in healthy volunteers is far from being settled, and even less so the question whether such effects are more pronounced after left or right hemispheric TMS interventions, thus either mimicking or contradicting the deficits seen in hemineglect patients.

Here, we assessed the functional relevance of left and right parietal cortex in attention control, as measured by LB and LM tasks, in a meta-analysis of all existing TMS studies. We explicitly wanted to aggregate all the studies targeting either the left hemisphere or right hemisphere or both to evaluate: (1) whether right or left PPC TMS could indeed induce an attentional bias on landmark/line bisection task, (2) whether there is any difference of effect size between those two hemispheres, and (3) whether this pattern between left and right hemisphere is similar to what is typically observed in neglect patients.

## Methods

### Study Selection

The current review was not registered but followed the PRISMA guidelines. The literature search was conducted on PubMed, Web of Science, and Elsevier databases using the search codes: (TMS OR “transcranial magnetic stimulation”) AND (PPC OR “parietal”) AND (“landmark” OR “bisection” OR “spatial attention” OR “neglect”). The search was restricted to journal articles written in English, between 1994 and February 2022. Two researchers searched articles fully independently according to the PRISMA guideline and inconsistencies in the search results were resolved in team discussions.

### Inclusion and Exclusion Criteria

Search results were imported into Endnote and passed through three screening rounds. Duplicates were removed in the first screening round. The two researchers conducted the abstract and full-text review in the second and third rounds. The inclusion criteria were: (1) at least 5 healthy human participants, (2) TMS targeting any regions of the PPC, (3) using the LM and/or LB tasks, and (4) comparison between active and baseline conditions. Baseline conditions could be in the form of no TMS, sham TMS, or stimulation over a control site (e.g., the vertex). Studies were excluded if they used a non-TMS stimulation technique, for example, transcranial alternating current stimulation (tACS) or transcranial direct current stimulation (tDCS), or did not involve any stimulation.

We only considered studies using inhibitory/disruptive TMS protocols. The inhibitory/disruptive TMS protocols included offline repetitive TMS (rTMS) at a low frequency (less than 5 Hz), online event-related TMS(ER), single/paired pulse TMS (SP/PP), and continuous Theta Burst Stimulation (cTBS).

### Data Extraction and Management

One researcher used a standardized data extraction form specifically designed for this review to collect data from the included studies. Extracted data included the following: author, publication year, description of participant sample (age, sex, handedness), task used, and TMS related parameters (frequency, offline/online, duration, stimulation site, stimulation type, and baseline condition). Results were extracted in terms of statistical values reported for the following categories: left stimulation and ipsilateral bias, left stimulation and contralateral bias, right stimulation and ipsilateral bias, and right stimulation and contralateral bias. For articles only reporting data in figures, numerical results were extracted from the figures using the GetData Graph Digitizer 2.24. All of these extraction steps were double-checked by other researchers.

### Statistical Analysis

Statistical analyses were performed with SPSS (IBM), Stata, and RStudio (Metafor and Meta package). Changes in detection performance in participants were analyzed using paired or one sample *t*-tests. For studies that reported the *F* value, the formula *t* = √*F* was used to estimate the *t*-test statistic from the one-way analysis of variance (Lipsey & Wilson, 2001). For studies reported mean value and standard error of baseline and TMS conditions, rather than *t*-statistic, we used the paired sample *T*-statistic and formulas outlined in Morris and DeShon (2002) to derive the correlation between outcome measures.

1 $$\frac{\underset{\_}{{X}_{1}} - \underset{\_}{{X}_{2}}}{\sqrt{\frac{{S}_{1}^{2} ({n}_{1} - 1) + {S}_{2}^{2} ({n}_{2} - 1)}{{n}_{1} + {n}_{2} - 2}}} = \frac{\underset{\_}{{X}_{1}}-\underset{\_}{{X}_{2}}}{{S}_{\mathrm{pooled}}}$$

2 $${S}_{\mathrm{pooled}}= \frac{{S}_{\mathrm{gain}}}{\sqrt{2(1-r)}}$$

*X* = mean value from baseline (*X*_1_) and experimental (*X*_2_) conditions; *s*^2^ = variance for the baseline (*s*_1_) and experimental (*s*_2_) conditions; *n* = total sample size; *s*_gain_= standard deviation squared (experimental condition); *r* = correlation between baseline and experimental conditions.

The effect sizes in the form of Cohen’s *d* were then calculated in these studies to characterize the difference in performance between the baseline (control) condition and each TMS condition, including studies using rTMS, single/paired pulse, cTBS, and event-related triggered stimulation. The current Cohen’s *d* was coded as the ipsilateral shift effect, independently of whether TMS stimulated left or right hemisphere. For studies that reported *t* or *F* value, effect size: *t*_to_*d* function in “Meta” package was used to get the Cohen’s *d*. For studies that reported data as mean value and standard error, the escalc function was used to obtain the (bias-corrected) standardized mean differences and corresponding sampling variances and transformed it to Cohen’s *d* according to the “Metafor” package. Since few of these studies reported the change of detection performance (compared with baseline, baseline as 0 or 1), then Stata software was used to get the corresponding *t* value.

The cumulative effect size was determined by weighting the effect sizes of each study by the inverse of their variance (i.e., precision). The weights were then summed and divided by the sum of the weights to obtain the overall effect size (fixed effects model) or also take into account the between-study heterogeneity (random effects model). In order to support the choice between fixed or random effects models, heterogeneity was quantified with a *Q* test. A forest plot was generated to visualize Cohen’s *d* by study. Next, a funnel plot was generated to visualize publication bias, further supported using an Egger test. This full process was repeated in a subgroup analysis of stimulation hemisphere (RPPC vs. LPPC). Finally, a meta-regression was performed to ensure other factors like publication year, and methodological factors (including control conditions, online/offline protocol, targeting by 10–20 system or MRI, comparison based on pre-post or post only; note all these factors were transferred to dummy variables) did not affect results, along with a sensitivity analysis to confirm that no single study exerted too much influence over the conclusion of the meta-analysis.

### Multilevel Meta-Analysis

For studies include multiple interventions in one sample, dependence might be introduced. Given statistical independence was one of the core assumptions of meta-analytic pooling (Harrer et al., 2021). A dependency between effect sizes (i.e., the effect sizes are correlated) might artificially reduce heterogeneity and then lead to false-positive results. These dependencies that may exist in these nested designs can be handled using a multilevel meta-analysis. In multilevel meta-analysis, the variance in observed effect sizes is decomposed into sampling variance (level 1), between-study variance (level 2) and variance between groups of studies (level 3), and the moderating effect of characteristics of studies (at level 2) and groups of studies (at level 3) can be explored. This analysis was conducted using the “Metafor” package in *R*.

### Bayesian Meta-Analysis

In addition to the conventional meta-analysis outlined above, we also performed a Bayesian meta-analysis. Within the *R* statistical computing environment, we used the “brms” package (Burkner, 2017), based on the Stan software (Carpenter et al., 2015), to fit Bayesian multilevel models. The first step of Bayesian analysis was defining a prior distribution of standardized mean difference (SMD) as SMD ~ N (0, 1) and heterogeneity (*τ*) as *τ* ~ HC (0, 0.5) then set up the formula for the model and the MCMC algorithm run 4000 iterations to fit the model. Before evaluating model fit, convergence was assessed by posterior predictive checks and *R*-hat values of the parameter estimates. Based on the obtained Bayesian model, we calculated the exact probabilities that the meta-analytic effect will be smaller/larger than a given effect size value by looking at the empirical cumulative distribution function (ECDF) of the posterior distribution for the pooled effect size.

## Results

### Overview

**Fig. 1:**
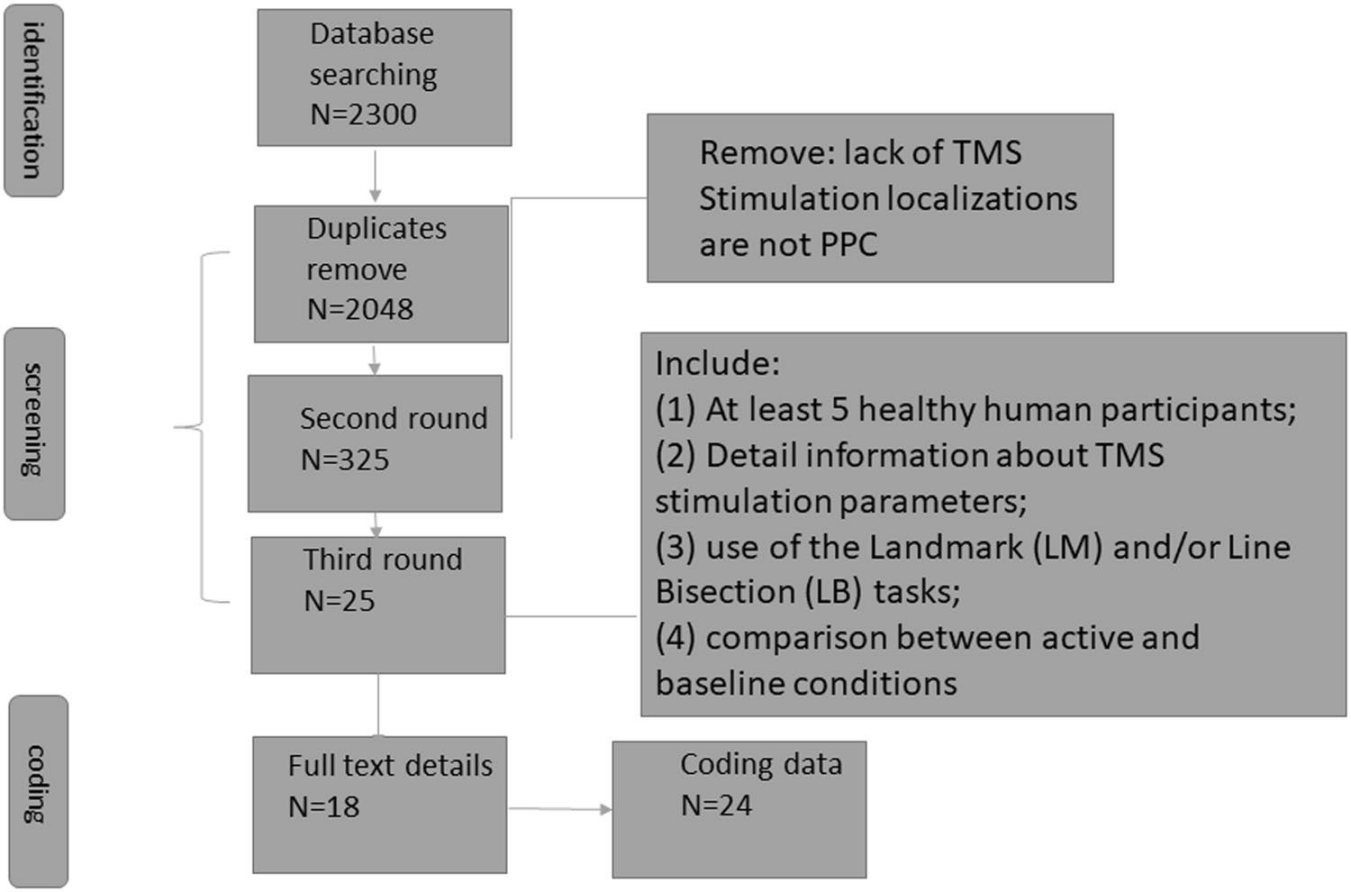
Flowchart of data extraction including database searches, the screening of study abstracts and full-texts, and the reasons for excluding studies

The initial literature search resulted in 2048 articles (duplicates removed) of which 325 were included in the related full-text review (details in Fig. 1). A total of 24 datasets from 18 different studies met the including criteria. Stimulation parameters and participants’ information are shown in Table 1.

**Table 1:** Summary of all included studies

Study	Participants	Age	Handedness	Task	TMS protocol	Type of TMS	Localization	Baseline	Pre-post
Bagattini et al., 2015	20 (13 females)	19–34	Right	LB	Offline, 90% of RMT, last for 30 min at 1 Hz	rTMS	10–20 system coordinates P4–P8	No TMS	Pre-post
Bjoertomt et al., 2002	6(4 females)	21–26	Right	LB	Online, 65% of MSO, at stimulus onset, last for 500 ms each trial	SP	rPPC by TMS(behaviorly hunting procedure)and structural MRI co-register check	Sham	Post only
Brighina et al., 2002	11	28–68	Right	LM	Online, 115% RMT, at stimulus onset, 10 pulses per trial at 25 Hz	ER	10–20 system coordinates P6	Sham	Post only
Cazzoli & Chechlacz, 2017	24 (12 females)	26.5 ± 5.1	17 right and 7 left	LB	offline, 80% RMT, 801 pulses, 3-pulses bursts at 30 Hz	cTBS	MRI localizer on rIPS and lIPS	Sham	Pre-post
Ellison et al., 2004	5 (2 females)	21–36	Right	LM	Online, 65% of MSO, at stimulus onset, 5 pluses per trial at 10 Hz	ER	3 × 3 cm grid hunting rPPC	Sham	Post only
Fierro et al., 2000	11	25–67	Right	LM	Online, 115% RMT, at stimulus onset 10 pulse per trial at 25 Hz	ER	10–20 system coordinates P5, P6	Sham	Pre-post
Fierro et al., 2001	10	20–68	Right	LM	Online, 115% RMT,(150 ms, 225 ms, 300 ms) after stimulus onset, 10 pulses per trial at 25 Hz	SP	10–20 system coordinates P6	No TMS	Post only
Fierro et al., 2006	13	24–30	Right	LM	Online, single pulse,120% RMT, 150 ms after stimulus; paired-pulse,150 ms after stimulus	SP and PP	10–20 system coordinates P6	no TMS	Post only
Ghacibeh et al., 2007	10 (5 females)	21.8 ± 5.2	Right	LB	Online,15% of MSO above the RMT, at “go” instruction onset, last for 5 s trains at 5 Hz	ER	10–20 system coordinates P6	No TMS	Pre-post
Giglia et al., 2015	15 (8 females)	28.2 ± 6.3	Right	LM	Online, 110% RMT, 100 ms before visual task, 5 pulses per trial at 10 Hz	ER	10–20 system coordinates P6	No TMS	Post-only
Mahayana et al., 2014	15(5 females)	23	NA	LM	Online, 60% of MSO, at stimulus onset, 5 pluses per trial at 10 Hz	ER	Individual MRI scans on rPPC	No TMS	Post-only
Oliveri et al., 2009	7 (3 females)	20–36	NA	LB	Offline, 90% RMT, last for 10 min at 1 Hz	rTMS	P4 (RH), EEG 10–20	No-TMS	Post-only
Oliveri & Vallar, 2009	10 (9 females)	21–34	Right	LB	Online, 100% RMT, at stimulus onset, 10 pulses per trial at 25 Hz	ER	P4 and 1.5 cm anterior to P4—EEG 10–20	Sham	Post-only
Salatino et al., 2019	13 (9 females)	26.77	Right	LM	Online, 115% of RMT, at stimulus onset	ER	3 × 3 cm grid hunting rPPC	Sham	Pre-post
Salatino et al., 2014	8 (5 females)	21–28	Right	LM	online,115% of RMT,150 ms after stimulus onset,	SP TMS	3 × 3 cm grid stimulation P5 and P6 as the stimulation center; EEG 10–20)	No-TMS	Pre-post
Szczepanski & Kastner, 2013	6 (2 females)	26–38	Right	LM	Online, 60% of MSO, 200 ms after stimulus onset, 10 pulses per trial hz	SP	Topographic ROIs overlaid on individualized MRI (right and left IPS1/2)	No-TMS	Pre-post
Schintu et al., 2021	17 (11 females)	25.94 ± 1.01	Right	LB	Offline, 80% RMT, 600 pulses, 3-pulse bursts at 50 Hz	cTBS	MRI-guided, Brainsight frameless stereotaxic system	No-TMS	Pre-post
Mariner et al., 2021	14 (9 females)	18–30	Right	LB	Offline,70% RMT, 600 pulses, 3-pulses bursts at 50 Hz	cTBS	10–20 system coordinates P4	No-TMS	Pre-post

*RMT* resting motor threshold, *MSO* maximum stimulator output, *rTMS* repetitive TMS, *SP* single pulse, *PP* paired-pulse, *ER* event-related, *cTBS* continuous theta burst stimulation, *MRI* magnetic resonance imaging, *EEG* electroencephalogram

As illustrated in Fig. 1, from the third round screening to the coding stage, 7 studies were excluded because they did not report the actual TMS effect on the attention shift but an interaction effect with other variables (such as Cattaneo et al., 2009) or they used excitatory TMS (Kim et al., 2005) rather than inhibitory protocols. Within the final 18 studies, some conducted both right and left hemisphere stimulation and are thus represented as two data points (such as Cazzoli & Chechlacz, 2017; Szczepanski & Kastner, 2013). Two studies (Bjoertomt et al., 2002; Ellison et al., 2004) used the landmark task but reported the attention shift by condition, such as right elongated line/bisected line/left elongated line and left side shorter/right side longer. For these two studies, conditions were combined by formula (3), (4), and (5) to obtain average sample size, mean value, and standard deviation so that found average effect as previous studies (Higgins et al., 2019). For the Fierro studies (Fierro et al., 2000, 2001, 2006), the single or paired pulse time intervals were excluded. Some studies included additional experimental manipulations of task-related factors such as distance to the monitor, the eccentricity of the stimuli, or additional TMS targets other than P3/P4. In these cases, we opted to include those conditions that were most similar to the other studies included here. Specifically, in the studies of (Bjoertomt et al., 2002; Giglia et al., 2015; Mahayana et al., 2014), where several viewing distances from the display (near vs. far /vs. far with stick) were reported, only near stimuli were included here since 60 cm distance was common in other studies. In the studies of Salatino and her colleagues (Salatino et al., 2019; Salatino et al., 2014), a 3 cm × 3 cm target grid was centered over P3 or P4 according to the 10–20 EEG system. Although the P5 and P6, as they reported, showed the highest effect in those 3 × 3 targets, P3 and P4 were included instead to ensure optimal comparison of TMS localization across studies.

3 $$\underline{N} = {N}_{1}+{N}_{2}$$

4 $$\underline{M}= \frac{{N}_{1}{M}_{1}+{N}_{2}{M}_{2}}{{N}_{1}+{N}_{2}}$$

5 $$\underline{SD}= \sqrt{\frac{({N}_{1}-1){SD}_{1}^{2}+({N}_{2}-1){SD}_{2 }^{2}+\frac{{N}_{1}{N}_{2}}{{N}_{1}+{N}_{2}}({M}_{1}^{2}+{M}_{1}^{2}-2{M}_{1}{M}_{2})}{{N}_{1}+{N}_{2}-1}}$$

*N* = combined sample size from conditions (*N*_1_, *N*_2_); *M* = combined mean value from conditions (*M*_1_, *M*_2_); *SD*^2^ = variance for the conditions (*SD*_1_^2^, *SD*_2_^2^); *SD* = combined standard deviation.

### Meta-Analysis of TMS-Induced Attention Shifts (TMS vs. Baseline)

To assess the TMS-induced attention shifts in general, 24 datasets were merged to yield a pooled effect size regarding left or right PPC stimulation. The test for heterogeneity was marginally significant (*I*2 = 33%; *τ*2 = 0.0361, *p* = 0.06) and we opted for the more conservative approach of proceeding with a random effects model, showing a significant (*z* = 7.14, *p* < 0.0001) positive Cohen’s *d* 0.58, 95% CI ranged from 0.42 to 0.74.

**Fig. 2:**
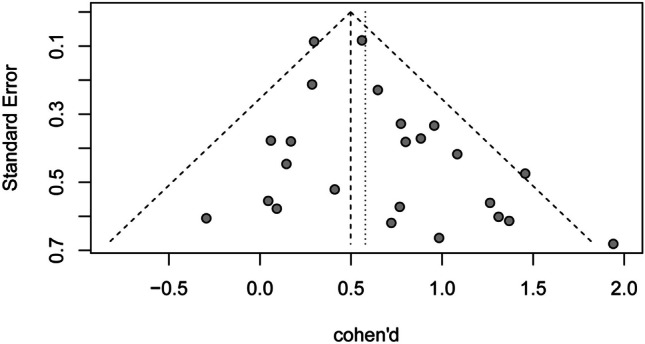
Funnel plot standard errors by standard difference in means

The Egger test showed a significant (*p* = 0.05) publication bias, visualized in the funnel plot (see Fig. 2).

To ensure that publication year and methodological factors did not affect results and no single study exerted too much influence over the conclusion of the meta-analysis, meta-regression and sensitivity analysis were conducted. Results showed that the publication year did not impact the effect (*p* = 0.19), control condition (*p* = 0.92), online or offline protocol (*p* = 0.35), TMS targeting by 10–20 system or MRI (*p* = 0.86) and the effect remained after omitting any single study. In sum, the overall TMS effect was convincing and robust.

As mentioned above, given the potential dependencies caused by multiple datasets stemming from single studies, we took such dependencies into account by integrating a third layer into the structure (we used a 3-level model including the sampling variation for each ES (level 1), variation across ESs within a study (level 2), and variation across studies (level 3)). Here, multiple separate datasets from two studies (Bjoertomt et al., 2002; Ellison et al., 2004), which collected data from multiple sites, were added. The full model showed that the pooled effect size was 0.68, 95% CI ranged from 0.43 to 0.99. After checking the variance distribution of the full model, it was clear that layers 1, 2, and 3 accounted for 47%, 36%, and 17% of the variance, respectively. The comparison between the full model and the leave-level 2-out and leave-level 3-out model suggested the full model as winning model (lower AIC and BIC level 2: *p* = 0.2; level 3: *p* = 0.55).

### Subgroup Comparison (LPPC vs. RPPC Stimulation)

**Fig. 3:**
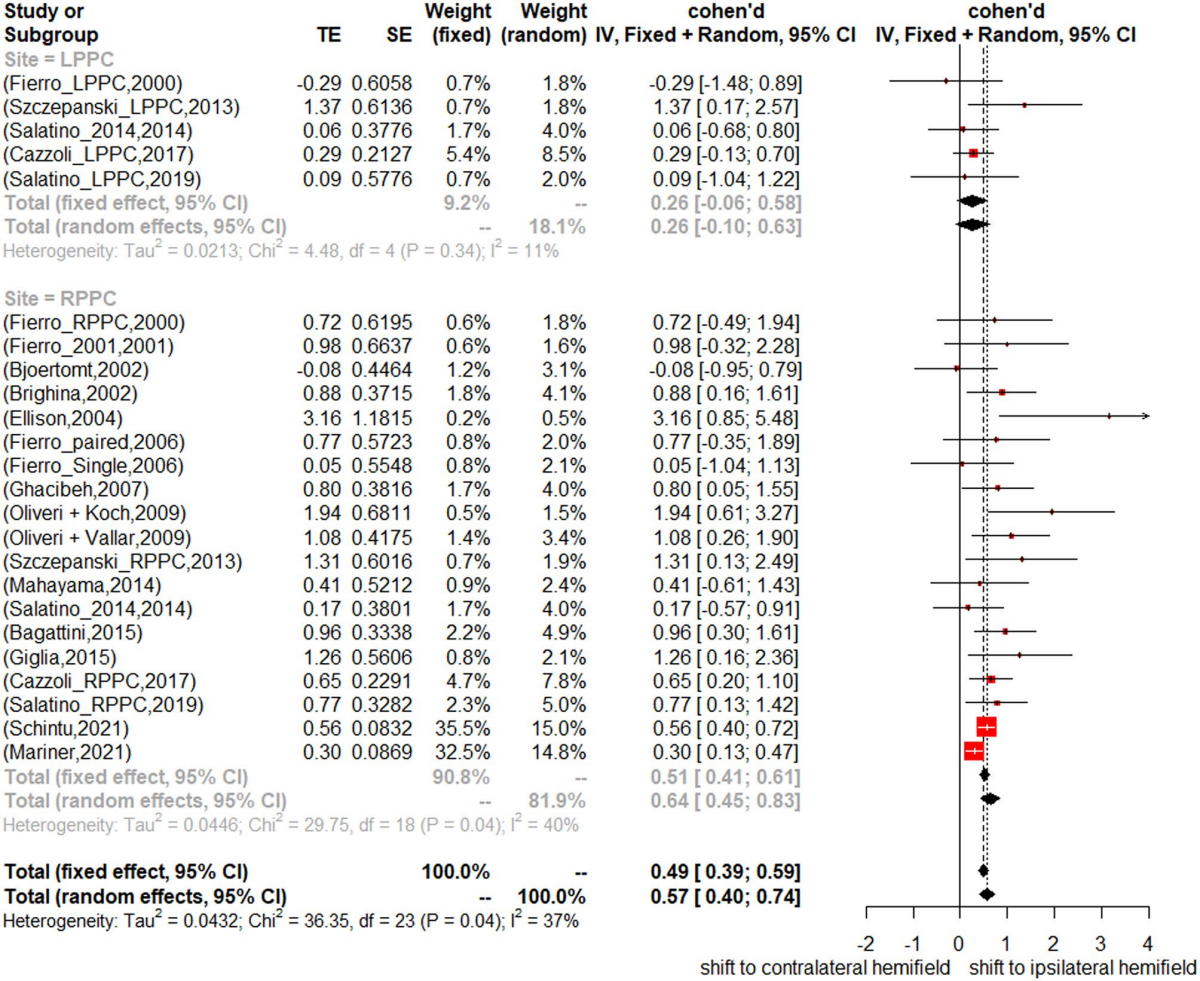
Subgroup comparison of the mean effect size (Cohen’s *d*) and 95% confidence intervals for the 24 datasets for line bisection/landmark performance change after left vs. right PPC stimulation

First, we assessed a potential “subgroup effect” of the LM and LB tasks. No significant difference between these two tasks was found (*Q* = 0.25, *df* = 1, *p* = 0.62). Then, given that most studies did not assess hemispheric asymmetries directly, we used subgroup analysis to test potential differences for results stemming from left PPC or right PPC TMS. The results showed that right PPC and left PPC stimulation did not cause significant different attention shift effects (*Q* = 3.5, *df* = 1, *p* = 0.06). To provide a more comprehensive understanding of the current data and explore potential patterns or trends, further analyses were conducted. The specific RPPC and LPPC effects are shown in Fig. 3. For the RPPC stimulation, the fixed effect model showed a significant positive Cohen’s *d* 0.52, 95% CI ranging from 0.42 to 0.62. For LPPC stimulation, the random effects model showed an insignificant positive Cohen’s *d* 0.26, 95% CI ranging from − 0.06 to 0.58. Because of the imbalance between LPPC and RPPC studies, a subgroup analysis containing 5 studies with both stimulation sites was conducted. Results showed the same pattern as the analysis of the entire dataset (The test for heterogeneity was not significant (*I*2 = 12.4%; *τ*2 = 0.001, *p* = 0.33), overall attention shift effect size: 0.4; 95% CI: 0.03 to 0.76; RPPC attention shift effect size: 0.64; 95% CI: 0.34 to 0.95; LPPC attention shift effect size: 0.08; 95% CI: − 0.42 to 0.58; and significant difference between LPPC and RPPC with *p* = 0.05). The subgroup analyses of the multilevel model were considered as well, the specific RPPC and LPPC effects were again different from each other. (For RPPC, shift effect size is 0.76, 95% CI is 0.32 to 1.19; for LPPC, shift effect size is 0.05, 95% CI is − 0.38 to 0.49.)

To ensure that the publication year and methodological factors did not affect results and no single study exerted too much influence over the conclusion of the meta-analysis, meta-regression and sensitivity analyses were conducted on RPPC datasets only because of the limited number of LPPC dataset. Results showed that the publication year (*p* = 0.07), control condition (*p* = 0.25), online or offline protocol (*p* = 0.26), TMS targeting by 10–20 system, or MRI (*p* = 0.90) did not impact the effect, and the effect remained after omitting any single study. In sum, the RPPC TMS effect was convincing and robust.

### Bayesian Meta-Analysis of RPPC and LPPC Stimulation (TMS vs. Baseline)

Bayesian meta-analyses have a similar aim as frequentist meta-analytic techniques but use a different statistical approach that is particularly suitable if the number of included studies is small (Harrer et al., 2021) or like in our case if the unbalanced number of studies investigating left PPC and right PPC may affect the robustness of the analyses. Bayesian meta-analysis is well-equipped to deal with this problem since it allows to directly make predictions in the estimation of between-study heterogeneity (Harrer et al., 2021). The Bayesian meta-analysis was separately applied to the RPPC and LPPC stimulation sample. After confirming convergence (*Ȓ* = 1), results showed nearly the same pooled effects for both hemispheres as compared to the results presented above (RPPC: a significant positive Cohen’s *d* 0.65, 95% CI ranged from 0.44 to 0.94; LPPC: an insignificant positive Cohen’s *d* 0.22, 95% CI ranged from − 0.27 to 0.69). The ECDF function showed that the probability of the pooled effect being greater than 0.4 is very high (96.2%) after RPPC disruption. Therefore, the attention shift effect after the RPPC stimulation is very likely to be meaningful. However, the probability of the pooled effect being greater than 0.4 after LPPC stimulation is very low (21%), which means the attention shift effect after the LPPC stimulation is not very likely to be obviously significant.

## Discussion

This meta-analysis study aimed to examine the hemisphere-specific effects of inhibitory TMS targeted at either right and/or left posterior parietal cortices on attention bias as measured by line bisection and landmark tasks. The main aim of this meta-analysis was to reveal whether TMS is capable of reliably producing significant spatial attention effects in healthy volunteers and whether such effects are more pronounced after left or right hemispheric TMS suppression. Based on the here presented data, we report quantitative evidence supporting the concept of functional lateralization with specifically right hemispheric TMS-induced spatial attention deficits in healthy participants.

The results across 18 high-quality studies highlight that inhibitory TMS on posterior parietal cortex can indeed induce attention biases as measured by line bisection and landmark tasks, which provide strong support for the functional role of PPC in the here assessed attention processes. Critically, there was only a significant attention shift effect induced by right, but not left, parietal TMS although the direct statistical comparison between the left and right hemisphere failed to reach significance. This pattern of results suggests a hemispheric asymmetry in the functional relevance of left versus right posterior parietal cortex that mimics and reproduces the hemispheric asymmetry seen in neglect patients after left versus right hemispheric lesions (Beis et al., 2004; Corbetta et al., 2005; Suchan et al., 2012). The results of the Bayesian meta-analysis consistently support the significant attention shift effect induced only by right parietal TMS. Given the results of both traditional inferential and Bayesian statistical analyses, we provide strong evidence for the functional relevance of PPC in line bisection and landmark tasks. Regarding hemispheric asymmetries, the pattern of results suggests a difference in effect size between left versus right parietal TMS on spatial attention bias but statistical results were not unambiguous and a few considerations regarding sample size and methodology have to be kept in mind. Given the importance of hemispherical asymmetry, some methodological remarks need to be considered. Not surprisingly, since most of the previous studies (such as, Bjoertomt et al., 2002; Ellison et al., 2004) aimed to confirm the relevance of the right, but not left posterior parietal cortex for attention, the number of studies included in the left PPC subgroup is smaller than in the right PPC subgroup. This implies that our analyses regarding hemispheric asymmetry have less statistical power and might be influenced by methodological differences between left PPC and right PPC studies. For that reason, we conducted a subgroup analysis with the five studies that investigated both hemispheres. Importantly, these five studies had consistent results for right PPC but mixed results for the left PPC. To be specific, four (Cazzoli & Chechlacz, 2017; Fierro et al., 2000; Salatino et al., 2019; Szczepanski & Kastner, 2013) of the five studies reported an effect for right PPC stimulation whereas only one study (Szczepanski & Kastner, 2013) reported an effect for left PPC stimulation. For this subset of studies, it is very unlikely that methodological factors have contributed to the observed hemispheric asymmetry because each pair of left and right PPC effect sizes originates from the same experiment. Moreover, in addition to the results of the here reported traditional meta-analyses, also the separately conducted Bayesian meta-analysis came to the same conclusion of a much more likely significant effect following right as compared to left parietal TMS.

The current findings can be interpreted in different ways. First, on a very general level, the results can be seen as support for right hemispheric dominance in attention. However, regarding the opposing theories of attention, such stronger contralateral attentional effects after right hemisphere suppression do not necessarily imply right hemisphere dominance in the sense of a generally stronger contribution to attention per se as proposed by Heilman’s model (Heilman & Van Den, 1980) of spatial attention. The here reported hemisphere-specific functional deficits after right parietal TMS are just as much in accordance with predictions based on Kinsbourne’s model (Kinsbourne, 1977) according to which suppressive TMS over right parietal cortex leads to disinhibition of the (dominant) left parietal cortex resulting in increased bias towards the ipsilateral (right) side, and thus to left attention deficits. Moreover, instead of right or left hemisphere dominance, our findings may also simply imply that the right hemisphere is more susceptible to interference to some extent. In this sense, whereas the current meta-analysis provides support for asymmetric TMS attention effects after right as compared to left parietal TMS, thereby also mimicking the lateralization reported in hemineglect patients, this experimental data is nonetheless still limited in informing us about which of the two proposed theories is more likely to be correct. This is due mainly to a fundamental problem of both the line bisection and landmark task used in these studies. Both paradigms do not allow to segregate the exact differential contributions of each hemisphere for each hemifield specifically, which is indispensable when referring to the separate functional role of each hemisphere for hemisphere-specific attention biases, gains, and costs towards the ipsilateral versus contralateral side of space (Duecker & Sack, 2015).

There are a few general considerations and potential limitations to consider. First, the meta-analytic results presented here are discussed in the context of spatial attention theories, but we only included studies using the line bisection task and landmark task. While this allowed us to have a very homogenous dataset, it does pose a problem regarding the generalizability of our findings. At present, we cannot conclude that the pattern of results revealed in this meta-analysis holds across the entire range of spatial attention tasks. Second, we could identify a small publication bias that may have led to an overestimation of effect size. However, the distribution of effect sizes clearly shows that the overall effect size is not the result of publication bias alone. Third, we performed a meta-regression analysis in order to identify additional potential confounders or factors of interest but none of them explained any variance across studies. Lastly, our meta-analysis clearly reveals a lack of direct comparisons between left and right PPC stimulation. Future research should aim to reveal these dominance of the right or left hemisphere for attention by other study paradigms such as visual detection tasks or spatial cueing tasks, or we could focus on TMS combined with neuroimaging studies and set more comprehensive range of studies, employing standardized protocols and minimizing heterogeneity. This would enhance the reliability and applicability of the findings in the field.

## Data Availability

The authors confirm that all data generated or analyzed during this study are included in this article.
